# Time Reversal Fusion for ECG Reconstruction Before and After Adversarial Fine-Tuning

**DOI:** 10.3390/bioengineering13101207

**Published:** 2026-10-16

**Authors:** Jiaqi Liu, Kwok Tai Chui, Lap-Kei Lee, Hao Ran Chi, Tik Yung Yip

**Affiliations:** 1 School of Science and Technology, Hong Kong Metropolitan University, Hong Kong SAR, China; s1304012@live.hkmu.edu.hk (J.L.); lklee@hkmu.edu.hk (L.-K.L.); s1346260@live.hkmu.edu.hk (T.Y.Y.); 2 Instituto de Telecomunicações, University of Aveiro, 3810-193 Aveiro, Portugal; ytchr@av.it.pt

**Keywords:** electrocardiogram reconstruction, time reversal, generative adversarial network, ensemble learning, adaptive fusion, spectral fidelity

## Abstract

Temporal reversal preserves electrocardiogram (ECG) samples while changing their presentation to a reconstruction model. We evaluated forward/reverse (F/R) fusion against matched forward/forward (F/F) ensembles using twelve-lead, seven-second windows from the normal-labeled CODE-15% subset. Each seed block comprised two forward branches and one reverse branch, with paired initialization for the second forward and reverse branches. Reverse outputs were restored to the original time axis before fusion. Across ten seed blocks of a temporal convolutional model trained with *L*_1_ loss, equal F/R fusion reduced test mean absolute error (MAE) by 1.437% relative to equal F/F, improving every block. Local adaptive F/R fusion reduced MAE by 2.140% against the same baseline. A matched continuation experiment compared *L*_1_ training with least-squares adversarial fine-tuning across six seed blocks. After adversarial training, equal F/R retained a mean MAE reduction of 0.717%, improving all six blocks. Lower residual cross-moments were consistent with complementary reconstruction errors. Equal F/R also improved seed-averaged amplitude error, correlation and R-peak *F*_1_ across the original-input condition and eighteen noise settings. Boundary-excluded evaluation and symmetric-padding controls retained mean directional benefits. These results support temporal direction as a source of reconstruction diversity beyond ordinary ensembling, with benefits retained under adversarial training.

## 1. Introduction

The electrocardiogram (ECG) records cardiac electrical activity through waveforms whose amplitudes, relative timing and lead relationships carry diagnostic information. Recommendations for ECG acquisition and processing place particular importance on preserving waveform fidelity when signals are sampled and filtered [1]. Large annotated datasets have supported neural models for automated twelve-lead interpretation and reproducible comparisons of those models [2,3,4]. Reconstruction addresses a related signal-processing task: recovering a waveform from a learned representation while preserving the details that distinguish beats and leads. Compact representation learning, inference of unobserved leads and synthesis of new ECGs impose different input and target requirements [5,6,7]. A low average amplitude error can coexist with attenuated small deflections, altered peak timing or a mismatch in spectral content.

Learned ECG enhancement methods address waveform fidelity through their architectures and training objectives. Xiong et al. [8] combined wavelet processing with a denoising autoencoder, while Chiang et al. [9] used a fully convolutional autoencoder to reconstruct ECGs under different noise levels. More recent approaches combine convolutional and attention-based representations [10] or introduce adversarial training [11]. These methods learn waveform structure under different input and target requirements. Reconstruction of an observed window, recovery from noise and synthesis of a new signal each require evaluation against the intended target.

Generative adversarial networks (GANs) pair a generator with a discriminator that learns to distinguish observed and generated samples [12]. Their use in healthcare spans images and physiological signals, with data quality assessed through task-dependent criteria [13]. In ECG modeling, GANs have been used to synthesize training examples [14] and to reconstruct multiple leads from a limited input [15,16]. An adversarial term can also be combined with a sample-wise loss when the output must correspond to a particular input. The reconstruction term penalizes deviation from the designated target waveform, whereas the discriminator responds to learned waveform characteristics. Testing whether temporal-direction effects persist under this additional signal requires discriminator-guided reconstruction training and a control for the additional training budget.

Temporal direction offers another way to vary the learned mapping. Reversal is an invertible permutation of time samples: it preserves recorded amplitudes and lead identities but changes the orientation of local waveform features and their temporal context. Training on the reversed input and target, then reversing the output back, produces a second reconstruction on the original time axis. Reordering alone neither removes noise nor adds information. Any benefit depends on how a finite-capacity model learns the reversed mapping and must therefore be assessed through a controlled comparison.

The usefulness of a reverse branch also depends on how it is combined with a forward branch. Independently trained neural ensembles are an established baseline [17]. Their error depends on both the accuracy of each member and the relationship between their errors [18]. Comparing a two-model forward/reverse system only with one forward model would confound temporal direction with ensembling. We therefore use a two-forward ensemble with the same architecture and training budget as the principal control. Equal forward/forward (F/F) and forward/reverse (F/R) fusion isolate direction under a fixed combination rule. Adaptive F/F and F/R then test the additional benefit of learned weights.

Each branch uses a fixed 100-dimensional bottleneck and the same twelve-lead reconstruction architecture throughout the comparison. Ten paired seed blocks assess the repeatability of the initial *L*_1_-trained result. Six blocks subsequently undergo matched *L*_1_ continuation or adversarial fine-tuning from the corresponding saved branch checkpoints. We evaluate the reconstructions on patient-disjoint cohorts and under controlled noise exposure. Spectral measures distinguish preserved high-frequency signal power from high-frequency error; automated peak matching assesses timing agreement.

The study tests whether temporal directional fusion improves reconstruction beyond ordinary forward ensembling under equal, static, record-adaptive and local-adaptive weighting. Matched continuation experiments distinguish persistence of the directional benefit after adversarial training from improvements due to additional generator updates. Residual-error decomposition relates fusion gains to branch complementarity, and noise and spectral analyses characterize the accompanying changes in reconstruction quality.

## 2. Related Work

### 2.1. Learned ECG Reconstruction and Denoising

Bottleneck autoencoders learn compact codes by reconstructing inputs through a lower-dimensional representation [5]. Denoising autoencoders make corruption part of training: the model receives a perturbed input and learns to recover its reference [19]. Robustness results must be interpreted in light of this supervision. Training on ordinary recordings and testing with added noise differs from learning explicitly from corrupted/reference pairs.

ECG denoising often combines learned representations with conventional signal processing. Xiong et al. [8] used wavelet thresholding and an improved denoising autoencoder to address complementary parts of the contamination. Chiang et al. [9] examined fully convolutional reconstruction, retaining temporal structure through convolutional operations rather than relying entirely on dense mappings. Peng et al. [10] combined convolutional wavelet processing and a Transformer-based representation for denoising and classification. These approaches differ in how they retain local morphology and represent longer temporal context, and their reported performance depends on the training targets and noise protocols.

Other designs alter feature processing within the encoder and decoder. Singh and Sharma [20] used attention and skip connections for two-lead ECG denoising. Jhang et al. [21] combined pixel-shuffle operations with a pooling mechanism to address electrode-motion contamination. Lin et al. [22] separated signal and noise representations within a denoising autoencoder. Feature selection, resolution recovery and latent organization are architectural alternatives to changing the temporal orientation of otherwise matched models.

The present model uses a temporal convolutional network (TCN), with dilated residual convolutions to represent sequential context [23]. Its encoder retains a temporal feature grid before projection to a fixed latent vector. Holding the architecture constant allows us to compare temporal directions within a single model family, without introducing differences among convolutional, recurrent, and attention-based architectures. We introduce controlled noise only during evaluation to measure how temporal fusion changes reconstruction under original and perturbed inputs.

### 2.2. Generative and Adversarial ECG Modeling

ECG generation includes several distinct tasks. Dynamical models synthesize signals through an explicit description of beat morphology and heart-rate variability [24]. Personalized GANs have generated heartbeat examples for patient-specific classification [14], while simulator-based GANs incorporated a physiological simulator into adversarial synthesis [25]. Conditional diffusion models have also generated twelve-lead waveforms from diagnostic statements [26]. Reviews of ECG synthesis distinguish mathematical simulators from learned generative models and compare their evaluation practices [7,13]. Such generation does not require sample-by-sample reproduction of a particular observed waveform. Reconstruction requires correspondence to the supplied signal or a designated reference.

Adversarial reconstruction and denoising use this correspondence explicitly. Conditional and cycle-consistent GANs have been trained to recover reference ECGs from noise-contaminated inputs [11,27,28]. These designs include channel attention and, in Kim et al. [28], the modeling of skin-electrode impedance and device-related noise. Lead reconstruction uses different conditioning information. Lee et al. inferred precordial leads from limb leads, while Zhan et al. [15,16] used lead-I heartbeats and R-peak alignment for variable-duration twelve-lead reconstruction. Comparisons across lead reconstruction methods depend on both the selected input leads and the intended output [6]. Generative priors also support inverse problems: Bedin et al. used a diffusion-based heartbeat prior for artifact removal and morphological reconstruction from indirect signals [29]. Missing-lead inference, synthetic-data generation and same-input reconstruction require comparisons suited to their respective tasks.

Paired image translation has used reconstruction and adversarial objectives together [30]. The least-squares GAN (LSGAN) objective uses squared deviations of discriminator scores from real and generated labels [31]. We adopt this objective to fine-tune a waveform reconstructor, with a discriminator that operates on aligned twelve-lead waveforms without conditioning on a concatenated input–output pair. The comparison tests whether a reverse-trained branch retains its fusion benefit after adversarial training. A matched *L*_1_ continuation group distinguishes the effect of further generator training from the effect of the change in objective.

Distributional resemblance and fidelity to an individual target require separate assessment. Blau and Michaeli [32] formalized a perception–distortion trade-off for image restoration. That distinction is conceptually relevant here, although ECG evaluation requires waveform-specific measures. Research on synthetic ECG assessment uses both morphological and functional criteria [33]. We report amplitude, spectral and peak agreement separately for adversarial reconstruction.

### 2.3. Temporal Order and Physiological Asymmetry

Sequence presentations can influence a learned mapping even when the transformation is invertible. In sequence-to-sequence translation, Sutskever et al. [34] found that reversing source sentences improved encoder–decoder training. They did not reverse the target sentences; the result is therefore specific to that task and does not explain ECG reconstruction. An empirical survey of time-series augmentation similarly found that the benefits of transformations depend on the data and the model [35]. Whether reversal is beneficial or label-preserving must be assessed for the particular clinical task.

Temporal reversal has also been used in physiological waveform learning. Zhao et al. introduced a time reversal consistency strategy for remote photoplethysmography (rPPG), aligning predictions from forward and reversed facial-video sequences [36]. Their method regularizes predictions across temporal orientations. We train reconstruction branches independently on forward- or reversed-ECG targets and combine their aligned outputs without a cross-direction consistency loss. The matched two-forward ensemble tests whether directional pairing adds useful error complementarity.

Physiological temporal asymmetry concerns properties of the recorded process. Costa et al. [37] examined time irreversibility in heartbeat interval dynamics and its association with aging and disease, while Merino-Negrete et al. [38] studied lag irreversibility in ECG signals. Statistical comparisons have also shown that the suitability of an irreversibility measure depends on the underlying process [39]. These findings motivate examining temporal order but do not explain a reconstruction gain. We restore reverse outputs to their original orientation before evaluation, so the measured effect concerns the errors of independently trained models.

We apply reversal to both the reconstruction input and target. Aligning both back to forward time leaves the *L*_1_ reconstruction loss unchanged because reversal only permutes samples. Differences between the trained models arise from their learned mappings, optimization and architectural constraints, not from a numerical change in the target loss. An ordinary forward ensemble is therefore a direct control for temporal diversity.

### 2.4. Ensemble Diversity and Adaptive Fusion

Neural ensembles use different initializations, training samples or model structures to obtain distinct errors [40,41]. For regression, Krogh and Vedelsby [18] related ensemble accuracy to the accuracy and disagreement of its members. Liu and Yao [42] subsequently used an explicit negative-correlation penalty during joint ensemble training. Independently initialized deep ensembles obtain diverse predictions without an explicit correlation penalty [17]. Comparisons of classifier ensembles have shown that diversity measures cannot substitute for prediction accuracy [43]. We assess complementarity using both an exact reconstruction-error decomposition and the paired fusion result.

Mixtures of experts use a gating model to allocate input cases among constituent networks [44]. We fit the fusion head after freezing the reconstruction backbones, using only their aligned outputs. The weighting alternatives range from signal-independent per-lead weights to record-level and local weights, with local weights varying across lead and time. Applying each rule to both F/F and F/R separates the contribution of weighting from that of temporal direction.

The second forward branch and the reverse branch begin with paired initial weights and are trained independently, without a negative-correlation or consistency penalty. Replacing the second forward model with its reverse counterpart tests temporal complementarity under the same branch budget. An exact residual decomposition separates the contribution of second-branch error from the cross term between branch errors. It can therefore explain a fusion gain even when the reverse model is not more accurate on its own.

## 3. Materials and Methods

### 3.1. ECG Records and Patient Partitions

We used the publicly released CODE-15% dataset [45]. All 134,657 records in the preprocessed study cohort were linked to the official examination metadata and carried the normal ECG flag. This automatic annotation does not establish that every participant was clinically healthy. Each record contained twelve leads in the order I, II, III, aVR, aVL, aVF and V1–V6, sampled at 400 Hz. The stored windows contained 2800 samples, corresponding to seven seconds, and were used without further cropping.

The stored arrays were already on a normalized amplitude scale. Identity scaling was retained after a training-only range assessment of 512 records using the 1st and 99th percentiles. Inputs were clipped to [−1, 1] without additional fitted rescaling or per-record normalization. Every branch received the same stored signals, and amplitude errors are reported in normalized signal units, rather than millivolts. Conversion to millivolts requires the upstream amplitude transformation and its physical scale, which were not recoverable from the archived normalized windows.

Patient identifiers defined the training, validation and test partitions. Backbone training used 107,696 records from 87,698 patients. The development subset contained 2048 records from 1969 validation patients and was used for checkpoint and hyperparameter selection. The confirmation cohort contained 10,712 records from 8993 other validation patients. A further 775 validation records belonging to development patients were excluded from confirmation. The test cohort contained 13,426 records from 10,963 patients.

Fusion heads were fitted to in-sample backbone predictions from 8192 records representing 8046 patients in the training partition. No confirmation or test records were used for gradient updates or checkpoint selection. Aggregate reconstruction metrics and waveform examples from the confirmation and test cohorts were inspected during iterative development. These outcomes informed the final backbone design and the decision to examine local adaptive fusion. The backbone and fusion hyperparameters were retained, and the designated contrasts were fixed, before seven additional initial seed blocks were run. These repetitions assessed initialization variability within the same patient-disjoint cohorts rather than in a newly blinded patient evaluation.

### 3.2. Reconstruction Architecture and Temporal Alignment

The reconstruction network comprised a convolutional encoder, a 100-dimensional latent vector and a TCN decoder. Three kernel-7 convolutions mapped the 12 input channels to 64, 128 and 256 channels, with strides of 1, 2 and 2, respectively. Each convolution was followed by a leaky rectified linear unit (LeakyReLU). A 1 × 1 projection reduced the feature grid to eight channels. The resulting 8 × 700 representation was flattened and projected to the latent vector. This retained positional structure before the projection; global average pooling was not used.

The decoder projected the latent vector to 256 × 350 features. Eight residual blocks each used two kernel-7 convolutions with 256 channels, group normalization with eight groups [46] and Gaussian error linear unit (GELU) activations. Their dilation factors were 1, 2, 4, 8, 16, 32, 64 and 128. Three transposed convolutions increased the length from 350 to 700, 1400 and 2800 samples while reducing the 256 channels to 128, 64 and 32, respectively. A final kernel-7 convolution and hyperbolic tangent activation produced twelve leads. The network contained 17,432,024 trainable parameters. The residual decoder convolutions used causal padding, but the whole-window encoder, latent projection and normalization make the complete network noncausal.

A separate padding control used seed blocks 7–9 and replaced the causal padding in the residual decoder convolutions with symmetric padding. The architecture, parameter count, paired initialization, record order, preprocessing, absolute-error objective and 50-epoch budget were otherwise unchanged. Each block contained the same three branches as the causal comparison. Boundary analysis first reconstructed the complete 2800-sample window and then excluded 100, 200 or 400 samples from each end, retaining 2600, 2400 or 2000 samples, respectively. Separate left- and right-edge and 100-sample temporal-bin analyses characterized the temporal distribution of the gain. Cropped windows were not supplied to the network, and these analyses used the same set of confirmed and test patients.

Let *x* denote a real-valued 12 × *T* ECG window, where *T* = 2800. The lead index *l* runs from 1 to 12, and the time index *t* runs from 0 to *T* − 1. The reversal operator *R* acts only along time:

*R*(*x*)*_l_*_,*t*_ = *x_l_*_,*T*−1−*t*_ (1)

Each seed block contained forward branches *F*_A_ and *F*_B_ and a reverse branch *R*_B_. Their networks are denoted *G*_A_, *G*_B_ and *G*_R_, respectively. All outputs were aligned to the reference time axis as

*y_A_* = *G_A_*(*x*),  *y_B_* = *G_B_*(*x*),  *y_R_* = *R*(*G_R_*(*R*(*x*))) (2)

**Figure 1:**
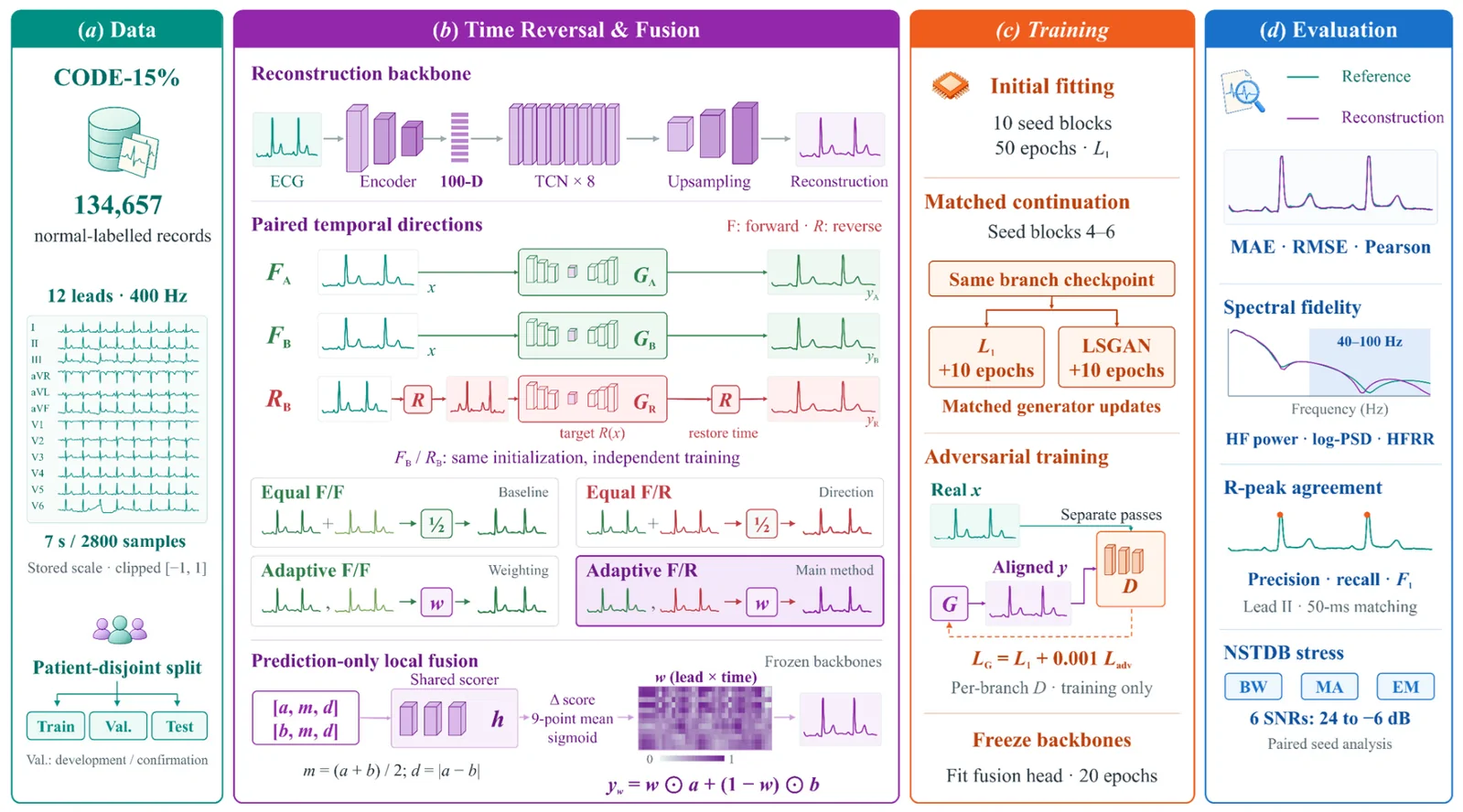
Experimental design for paired forward and reverse reconstruction, fusion ablations, matched continuation and waveform evaluation. The continuation panel depicts the original blocks 4–6; the same protocol was repeated in blocks 7–9.

The reverse network received the reversed input and reconstructed its reversed target. Its prediction was then reversed back to the original waveform orientation for fusion and evaluation. Lead order remained unchanged. We denote fusion of *F*_A_ with *F*_B_ as forward/forward (F/F), and fusion of *F*_A_ with the aligned *R*_B_ output as forward/reverse (F/R). These comparisons share *F*_A_ and differ only in the training direction of the second branch. Figure 1 summarizes the branches, fusion comparisons and continuation experiment. Reversal changes sample positions without altering their values or lead identities. Applying it twice restores the original samples exactly, without interpolation or filtering. This exact invertibility concerns the transformation and alignment; reconstruction fidelity remains a property of the learned model.

### 3.3. Backbone Training and Paired Controls

For the seed block *s*, *F*_A_ used initialization seed *s*, while *F*_B_ and *R*_B_ both used *s* + 10,000. The latter two branches began with identical weights and were trained independently. Data order and stochastic training seeds were matched within each block. The initial experiment used blocks 1–10, with 50 epochs per branch.

The reconstruction objective was the mean absolute error over all leads and samples, averaged across minibatch records:

(3) $$L_{\mathrm{rec}}\;=\;E_{x}[\frac{1}{12T}{\left\|y(x)\;-\;x\right\|}_{1}]$$

Here, *y*(*x*) is the aligned prediction, and the expectation is an empirical minibatch mean. The *L*_1_ norm sums the absolute entries of its argument. Reversal is an involutive permutation, so native reverse-target training and forward-aligned evaluation have the same reconstruction loss:

‖*G_R_*(*R*(*x*)) − *R*(*x*)‖_1_ = ‖*R*(*G_R_*(*R*(*x*))) − *x*‖_1_ (4)

Restoring the output orientation therefore leaves the reverse branch’s *L*_1_ objective unchanged, including during adversarial continuation.

Training used an Adam optimizer [47] with a learning rate of 10^−4^, momentum parameters (0, 0.999), a batch size of 64 and a gradient-norm clipping at 1. For each branch, we selected the trained checkpoint with the lowest development-set *L*_1_ loss. No discriminator was used in this initial stage. F/F and F/R each used two networks at inference, with the same total number of parameters and combined latent-vector capacity.

### 3.4. Equal and Adaptive Fusion

Equal F/F averaged *y*_A_ and *y*_B_, while equal F/R averaged *y*_A_ and *y*_R_. Let *a* and *b* denote the two aligned predictions to be combined, *m* their elementwise mean and *d* their absolute difference. A shared scorer *h* processed the concatenated channel inputs [*a*, *m*, *d*] and [*b*, *m*, *d*]. The local fusion weight was

*w*(*a*,*b*) = *σ*(*A*_9_(*h*([*a*, *m*, *d*]) − *h*([*b*, *m*, *d*]))) (5)

The operator *A*_9_ is a nine-sample moving average with replicate padding, and *σ* is the logistic sigmoid. Square brackets denote channel concatenation. The weight tensor has one value for each lead and sample. The fused prediction is

*y_w_* = *w*(*a*, *b*) ⊙ *a* + (1 − *w*(*a*, *b*)) ⊙ *b* (6)

The symbol ⊙ denotes elementwise multiplication. Exchanging *a* and *b* negates the score difference and complements their weights, leaving the fused prediction unchanged. At inference, the head receives only the two reconstructed waveforms, without the reference or original input.

The scorer used three kernel-9 convolutional layers with 32 channels and dilation factors 1, 4 and 16, followed by a twelve-channel 1 × 1 output projection. Its final projection was initialized to zero, giving initial weights of 0.5. The local and record-adaptive scorers each contained 29,292 parameters. Record-adaptive fusion replaced local smoothing with a temporal average before the sigmoid, giving one weight per record and lead. Static fusion learned twelve signal-independent per-lead logits. These alternatives were evaluated for both F/F and F/R, alongside the three individual branches.

Backbones remained frozen during 20 epochs of head training. Heads used *L*_1_ loss, Adam with learning rate 10^−3^ and momentum parameters (0.9, 0.999), batch size 64 and gradient clipping at 1. We selected the checkpoint with the lowest development MAE from the equal-weight initialization at epoch zero and all trained epochs.

Local adaptive F/R was the designated main method, and equal F/F was the principal baseline. Its comparison with an equal F/R isolated the additional effect of adaptive weighting, while its comparison with a local adaptive F/F isolated the temporal direction under the same weighting rule. These contrasts were fixed before the initial three seed blocks were expanded with seven further blocks.

### 3.5. Adversarial Fine Tuning and Matched Continuation

The *F*_A_, *F*_B_ and *R*_B_ checkpoints from blocks 4–9 were used to initialize matched *L*_1_ and GAN continuation groups. Each corresponding branch started from the same learned weights with reset optimizer states and received ten additional epochs, for 36 continuation runs. The generator learning rate was reduced to 10^−5^, with the other optimizer settings unchanged. Each run used 1694 batches per epoch and 16,940 generator updates in total. The GAN group also performed one discriminator update per batch. The groups therefore had equal numbers of generator updates, with additional computational cost in the GAN group.

Each GAN branch used its own twelve-lead waveform discriminator. Five kernel-7, stride-2 convolutions mapped the 12 input channels to 32, 64, 128, 256 and 256 channels, respectively, with LeakyReLU activations. A final kernel-3 convolution produced a local score map without a sigmoid. The discriminator contained 764,001 parameters and used Adam with learning rate 10^−4^ and momentum parameters (0, 0.999). Its input was the waveform alone; no input–output pairs were concatenated. Real and reconstructed waveforms, including reverse-branch outputs, were all presented in forward time.

Using least-squares adversarial objectives [31], the discriminator and generator losses were

(7) $$L_{D}=\frac{1}{2}E_{x,j}[{\left(D_{j}(x)-1\right)}^{2}+{D_{j}(\mathrm{sg}(y(x)))}^{2}]$$

(8) $$L_{G}=L_{\mathrm{rec}}+\lambda \;E_{x,j}[{\left(D_{j}(y(x))-1\right)}^{2}]$$

The index *j* identifies a position in the discriminator score map, and the expectation averages over records and score positions. The operator *sg*( ) stops the gradient, so predictions were detached during the discriminator update. During the generator update, discriminator parameters were frozen while gradients passed through its computation to the reconstructed waveform. The factor one-half was applied only to the discriminator loss.

We selected *λ* = 0.001 from 0.001 and 0.01 by development MAE in a two-epoch pilot on the seed-4 *F*_A_ branch. All 36 continuation runs started from the original source checkpoints, not the pilot weights. Generator selection considered trained epochs 1–10; the selected GAN generators came from epochs 5–10. Fusion heads were then fitted using the same frozen-backbone protocol, including the epoch-zero candidate. The discriminator was removed at inference. The system performs reconstruction of a supplied ECG, not unconditional sampling of a new patient waveform. Blocks 7–9 repeated the fixed protocol without another pilot or a change to the adversarial weight. Results are summarized for these three new blocks, the five nonpilot blocks 5–9 and the complete six-block set 4–9.

### 3.6. Controlled Input Noise

The stress cohort contained 2048 confirmation records, one per patient. Baseline wander, muscle artifact and electrode-motion recordings were taken from the MIT-BIH Noise Stress Test Database (NSTDB) [48,49], distributed through PhysioNet [50,51]. Noise was resampled to 400 Hz. For each ECG, one common noise segment and twelve independent segments were drawn. Each lead combined the common segment with its own independent segment using equal coefficients. The combined trace was mean-centered and normalized to unit root mean square (RMS).

For a signal-to-noise ratio (SNR) *ρ* in decibels and mean-centered reference-lead power *P*, the noise amplitude multiplier was

(9) $$\alpha =\sqrt{\frac{P}{10^{\frac{\rho }{10}}}}$$

Here, *P* is the mean squared deviation from the lead’s temporal mean; zero-power leads received no added noise. The SNRs were 24, 18, 12, 6, 0 and −6 dB. The three noise types and the original-input condition gave nineteen conditions in total. Each record received the same noise realization across methods, training seeds and continuation groups. Noise was added after input normalization and clipping, with no further clipping of the perturbed input.

Models and fusion heads were evaluated under these stress conditions without fitting or recalibration. The target was the corresponding original stored ECG, not an independently obtained artifact-free physiological recording. The protocol tests controlled twelve-lead perturbations constructed from recorded noise sources.

### 3.7. Reconstruction and Spectral Measures

MAE was the primary outcome. Root mean squared error (RMSE) was calculated within each record and then averaged across records. Pearson correlation was computed over time within each lead, averaged across the twelve leads and then across records. These are record-weighted summaries; patients with more than one evaluation record contribute those records separately.

R-peak agreement was evaluated in lead II using the same automatic detector for the reference and prediction. A third-order, zero-phase Butterworth filter retained 5–25 Hz. Polarity was selected from the filtered waveform’s 1st and 99th percentiles. Peaks required a separation of at least 300 ms, a height above the median plus 0.50 standard deviations (SDs), and a prominence of 0.35 SDs. Each reference peak was greedily matched to the nearest unused reconstructed peak within 50 ms. Precision, recall, *F*_1_ and matched counts were calculated per record and then averaged. Undefined precision or recall caused by an empty corresponding peak set was assigned zero; *F*_1_ was zero when precision and recall summed to zero. No independent human or gold-standard R-peak annotations were used. These scores measure agreement between automatic detections in the stored reference and reconstruction; errors shared by the detector can remain undetected by this comparison.

Power spectral density (PSD) was estimated by Welch’s method [52] with 256-sample Hann windows and 50% overlap. High-frequency (HF) prediction power and HF residual power were separately summed over the included 40–100 Hz bins, leads and records. Each sum was divided by the summed reference power in the same band within that seed’s cohort. The target ratios are one for prediction/reference power and zero for residual/reference power. We calculated these ratios within each seed before averaging across seed blocks.

Log-PSD mean squared error (MSE) was defined as the average squared difference between natural-log predictions and reference spectra, with 10^−12^ added before the logarithm. Errors were averaged over frequency bins within 0.5–100 Hz, leads and records. The frequency spacing was 1.5625 Hz; the 0.5 Hz lower bound defines bin inclusion, not spectral resolution. For the high-frequency residual ratio (HFRR), we subtracted a 61-sample moving average (152.5 ms) from the lead-II reconstruction residual. The RMS of this filtered residual was divided by the reference lead-II RMS. HFRR is distinct from the fixed-band HF residual power ratio.

### 3.8. Residual Complementarity and Statistical Analysis

Let *e*_A_ = *y*_A_ − *x* and *e*_Q_ = *y*_Q_ − *x*, where *Q* is *B* for F/F or *R* for F/R. For equal fusion, the cohort MSE satisfies

(10) $${\mathrm{MSE}}_{\mathrm{eq}}(A,Q)=\frac{1}{4}E[{e_{A}}^{2}]+\frac{1}{4}E[{e_{Q}}^{2}]+\frac{1}{2}E[e_{A}e_{Q}]$$

In this equation, the expectation averages all samples, leads and evaluation records within a seed. The cross term is an uncentered residual cross-moment. The MSE reduction produced by replacing *F*_B_ with *R*_B_ is

(11) $$\begin{array}{c} \Delta \mathrm{MSE}={\mathrm{MSE}}_{\mathrm{eq}}(A,B)-{\mathrm{MSE}}_{\mathrm{eq}}(A,R)\;\;\\ \;\;\;\;\;\;=\frac{1}{4}(E[{e_{B}}^{2}]-E[{e_{R}}^{2}])+\frac{1}{2}(E[e_{A}e_{B}]-E[e_{A}e_{R}]) \end{array}$$

The shared first-branch contribution cancels, and positive values in this decomposition favor F/R. By contrast, the statistical tables report comparison-minus-reference MAE differences, where negative values favor the first method. The decomposition uses cohort MSE, which differs from the mean record-level RMSE in the reconstruction tables.

Each metric was summarized by its mean and sample SD across paired seed blocks. Relative changes were calculated as ratios of seed-averaged condition means, not as averages of record-level or seed-specific percentages. The three designated initial MAE contrasts compared local adaptive F/R with equal F/F and with local adaptive F/F and equal F/R. Exact two-sided paired sign-flip tests were adjusted across these contrasts using Holm’s procedure [53]. Confidence intervals (CIs) were 95% *t* intervals for paired seed differences, without multiplicity adjustment for simultaneous coverage. Results were summarized for all ten blocks and, separately, for the overlapping subset of seven added blocks.

The six-block continuation experiment, the three-block padding control, and the noise and secondary-metric comparisons are descriptive. Repeated patients, leads and noise conditions were not treated as independent trained-model replications. Inference time was measured for the initial-protocol seed-1 models using resident 32-bit floating-point tensors on an NVIDIA H100 PCIe, with ten warm-up iterations and thirty synchronized repetitions at batch sizes of one and 64. Median batch latency and throughput included reversal and temporal realignment, where applicable. Data loading, transfer and host-side metric evaluation were excluded. Recorded training times sum the epoch-level wall-clock durations of training and development-set evaluation rather than the elapsed time of the parallel launcher.

## 4. Results

### 4.1. Directional Fusion and Weighting Ablations

Equal F/R reduced test MAE from 0.041493 for equal F/F to 0.040897, a 1.437% relative reduction, with lower MAE in all ten seed blocks. RMSE decreased from 0.067536 to 0.066772, and Pearson correlation increased from 0.960503 to 0.961300. Table 1 summarizes all eleven initial conditions. The single reverse branch *R*_B_ achieved a mean MAE of 0.043780, close to the two forward branches. The directional advantage was more consistent when the outputs were fused.

Static and record-adaptive F/R achieved mean MAEs of 0.040869 and 0.040847, respectively. Local adaptive F/R reached 0.040605, the lowest of the evaluated conditions. Relative to equal F/F, this was a 2.140% reduction, with a paired difference of −0.00088779 and 95% CI [−0.00118014, −0.00059543]. Compared with local adaptive F/F and equal F/R, the reductions were 1.331% and 0.713%, respectively. All three contrasts favored local adaptive F/R in every block, with Holm-adjusted *p* = 0.005859 as shown in Table 2.

The seven added blocks reproduced all three effects, with reductions of 2.187%, 1.310% and 0.752%. Each contrast improved in all seven blocks and had adjusted *p* = 0.046875. The paired comparisons separate the gain from temporal direction from the additional reduction achieved by local weighting under the 50-epoch backbone budget.

### 4.2. Directional Gains After Adversarial Training

Following adversarial fine-tuning, equal F/R achieved a test MAE of 0.039239, compared with 0.039523 for equal F/F. The mean reduction was 0.717%, with lower MAE in all six seed blocks. RMSE decreased from 0.064590 to 0.064265, and Pearson correlation increased from 0.963307 to 0.963698. The confirmation cohort showed a corresponding MAE reduction of 0.736%. Table 3 presents the continuation results together with the matched *L*_1_ group.

**Figure 2:**
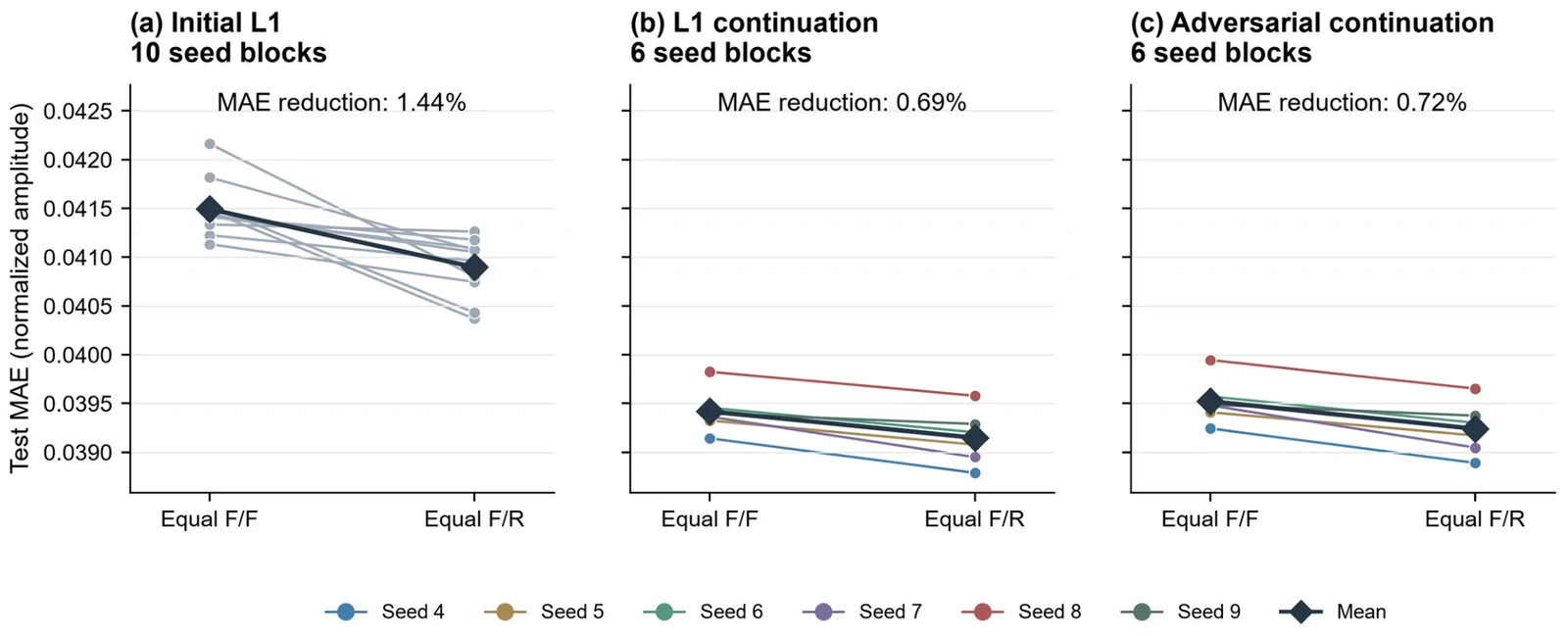
Paired test MAE for equal F/F and F/R fusion under (**a**) initial *L*_1_ training, (**b**) matched *L*_1_ continuation and (**c**) adversarial fine-tuning.

*L*_1_ continuation produced a similar directional reduction of 0.686%, from 0.039418 to 0.039148. For equal F/R, the *L*_1_ and GAN test MAEs were 0.039148 and 0.039239, respectively, a difference of 0.234%. Directional fusion thus remained beneficial within the adversarially trained models. Figure 2 compares the initial and continuation protocols. Thin lines connect each seed’s F/F and F/R values, while diamonds denote protocol means. The initial panel contains ten blocks, and each continuation panel contains blocks 4–9. The protocols are summarized separately.

The fixed-protocol replication in the three new blocks 7–9 retained GAN F/R MAE reductions of 0.708% on confirmation and 0.705% on test, with lower MAE in all three blocks. Excluding pilot block 4, the remaining blocks retained reductions of 0.691% and 0.681%, respectively, and had lower MAE across all five nonpilot blocks. These subsets assess initialization reproducibility on the previously examined patient cohorts.

Development MAE selected exact equal weighting (epoch zero) for all six F/R heads under both continuation objectives. Subsequent head updates reduced training loss without improving this selection score, so the selected local and equal F/R outputs coincide. Local F/F heads produced small changes. Table 4 reports these weighting effects together with the directional comparison and matched GAN-minus-*L*_1_ contrasts.

### 4.3. Complementary Residual Errors

In the GAN group, the mean residual cross-moment decreased from 0.004889 for F/F to 0.004782 for F/R. Equal-fusion MSE decreased from 0.005369 to 0.005310. Both changes occurred in all six seed blocks. The total MSE reduction of 0.000058574 comprised 0.000005418 from the second-branch error term and 0.000053156 from the residual cross term. *L*_1_ continuation showed the same pattern.

**Figure 3:**
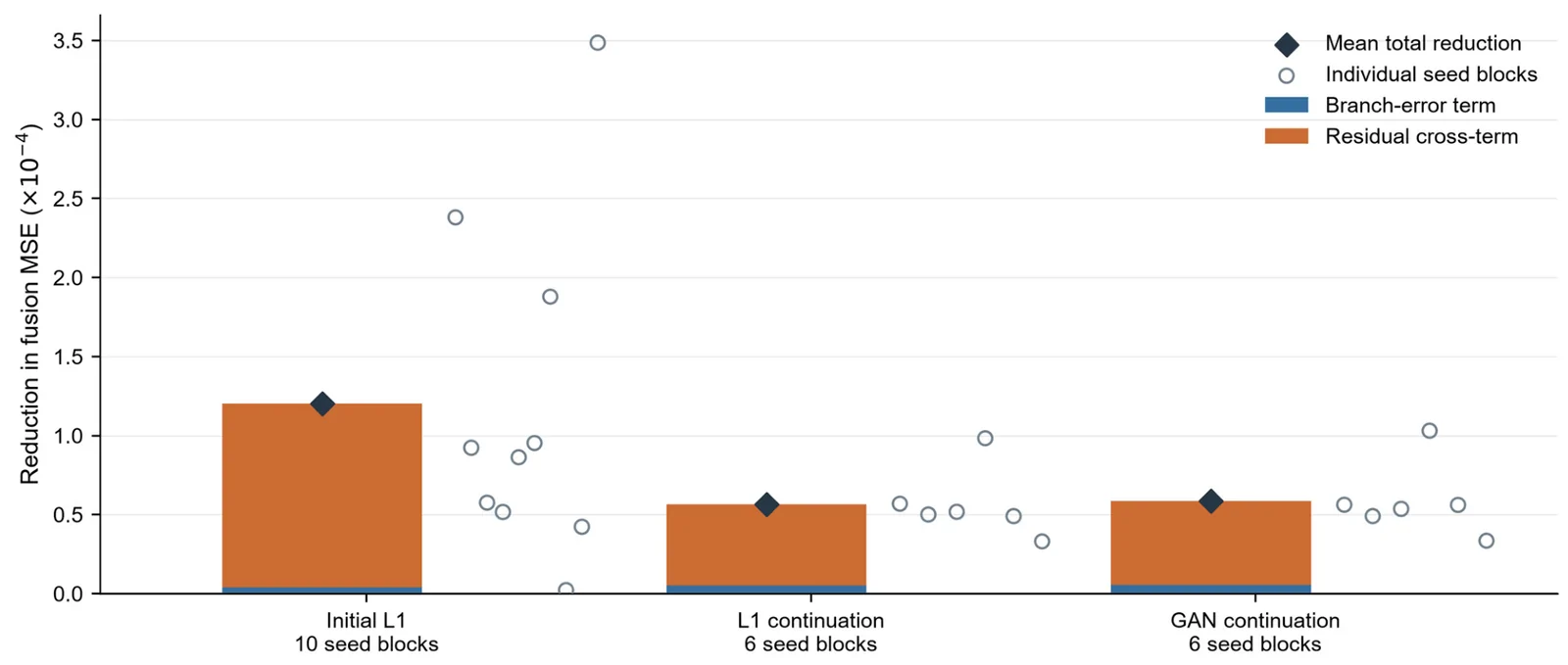
Branch-error and residual cross-term contributions to the MSE reduction from equal F/R fusion across the initial and continuation protocols.

Figure 3 displays the decomposition: stacked bars show the mean components, diamonds show their sum, and open circles show the seed-specific total reductions. The axis expresses squared normalized-amplitude values in units of 10^−4^, with positive reductions favoring F/R. The cross-moment quantifies uncentered error dependence in the decomposition. On average, the reverse branch reduced fusion error through both its own reconstruction error and its lower error cross-moment with the first branch. The cross-term contribution was positive in all six continuation blocks, including the two blocks in which the second-branch error contribution was negative.

### 4.4. Reconstruction Under Controlled Input Noise

After GAN continuation, equal F/R achieved lower seed-averaged MAE and RMSE and higher seed-averaged Pearson correlation and R-peak *F*_1_ than equal F/F in all nineteen stress conditions. At 0 dB, the MAE reductions were 2.912% for baseline wander, 2.205% for muscle artifact and 2.112% for electrode motion. At −6 dB, they were 4.228%, 2.580% and 1.438%, respectively. All six seeds had lower F/R MAE in eight of the nineteen conditions. Across the eighteen noisy conditions, the mean MAE reductions ranged from 0.692% to 4.228%; all eighteen retained a mean reduction when pilot block 4 was excluded.

Table 5 reports all prescribed noise/SNR conditions, including the initial direction and adaptation effects and matched GAN-minus-*L*_1_ comparisons. The original-input and noisy rows use the same 2048-record stress subset, not the full test cohort. In the initial experiment, local weighting improved on equal F/R at 24, 18 and 12 dB; equal weighting performed better at 6, 0 and −6 dB. The weighting benefit depended on the noise regime. Values are relative changes in MAE; parentheses give the number of seed blocks with lower MAE out of the total evaluated. Negative values favor the first method in each contrast.

**Figure 4:**
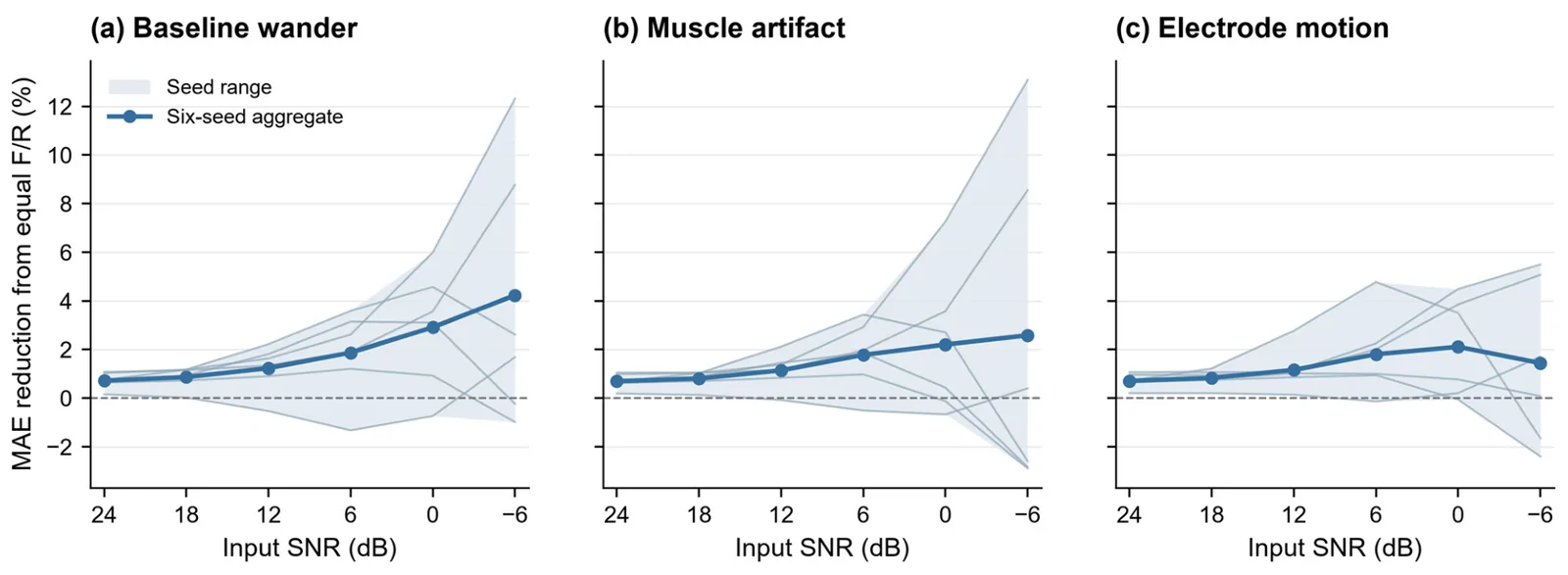
Relative MAE reduction from equal F/R compared with equal F/F after adversarial fine-tuning under (**a**) baseline wander, (**b**) muscle artifact and (**c**) electrode-motion noise. The gray lines represent individual seeds, whereas the dotted line indicates the 0% reference level.

Figure 4 shows all eighteen noisy GAN conditions. Solid curves give the relative reductions calculated from the ratio of seed-averaged MAEs. Thin curves show individual seeds, and the shaded envelopes span their minimum and maximum values, not confidence intervals. The wider ranges at −6 dB indicate greater seed variability under severe corruption. The GAN panels include all six continuation blocks.

At 24 and 18 dB, all six GAN blocks had lower F/R MAE for each noise type. At 12 dB, five of six improved for baseline wander and muscle artifact and all six for electrode motion; at 6 dB, five of six improved for each type. At 0 dB, baseline wander and electrode motion improved in five of six blocks and muscle artifact in four of six. At −6 dB, the corresponding counts were four, four and three for baseline wander, electrode motion, and muscle artifact. For −6 dB electrode motion, automated R-peak *F*_1_ increased from 0.797524 to 0.811757 in the six-block mean, with improvement in all six blocks; the nonpilot mean increased from 0.796098 to 0.808101, with improvement in all five. The stress results retain average direction and peak-agreement benefits while showing greater MAE variability under severe corruption; the prescribed SNRs do not define a clinical threshold.

### 4.5. Spectral Fidelity and Peak Agreement

The spectral measures distinguish retained signal power from residual error (Table 6). In the initial equal-fusion comparison, the mean HF prediction-to-reference ratio increased from 0.793657 for F/F to 0.814751 for F/R, while the HF residual-to-reference ratio changed from 0.507385 to 0.510848. Local adaptive F/R increased the prediction ratio further to 0.869847, with an error ratio of 0.531382. HFRR decreased from 0.057807 for equal F/F to 0.056787 for local adaptive F/R. Table 6 reports HFRR alongside the fixed-band spectral measures.

After continuation, equal F/R had prediction/reference power ratios of 0.866559 under *L*_1_ and 0.870552 under GAN training. The respective error/reference ratios were 0.482172 and 0.487049, with log-PSD MSE values of 10.701651 and 10.960397. The directional amplitude-error gain persisted under both continuation objectives; Table 6 reports their corresponding spectral profiles.

Equal F/R increased R-peak recall, *F*_1_ and the mean matched count (Table 7). Under the initial protocol, recall increased from 0.940903 to 0.941702 and *F*_1_ from 0.956151 to 0.956620 relative to equal F/F. Mean matched counts increased from 9.177581 to 9.189453 per record. After GAN continuation, *F*_1_ increased from 0.957651 to 0.957873 and matched counts from 9.210003 to 9.214435. Precision decreased slightly in the initial comparison and increased slightly after GAN continuation.

**Figure 5:**
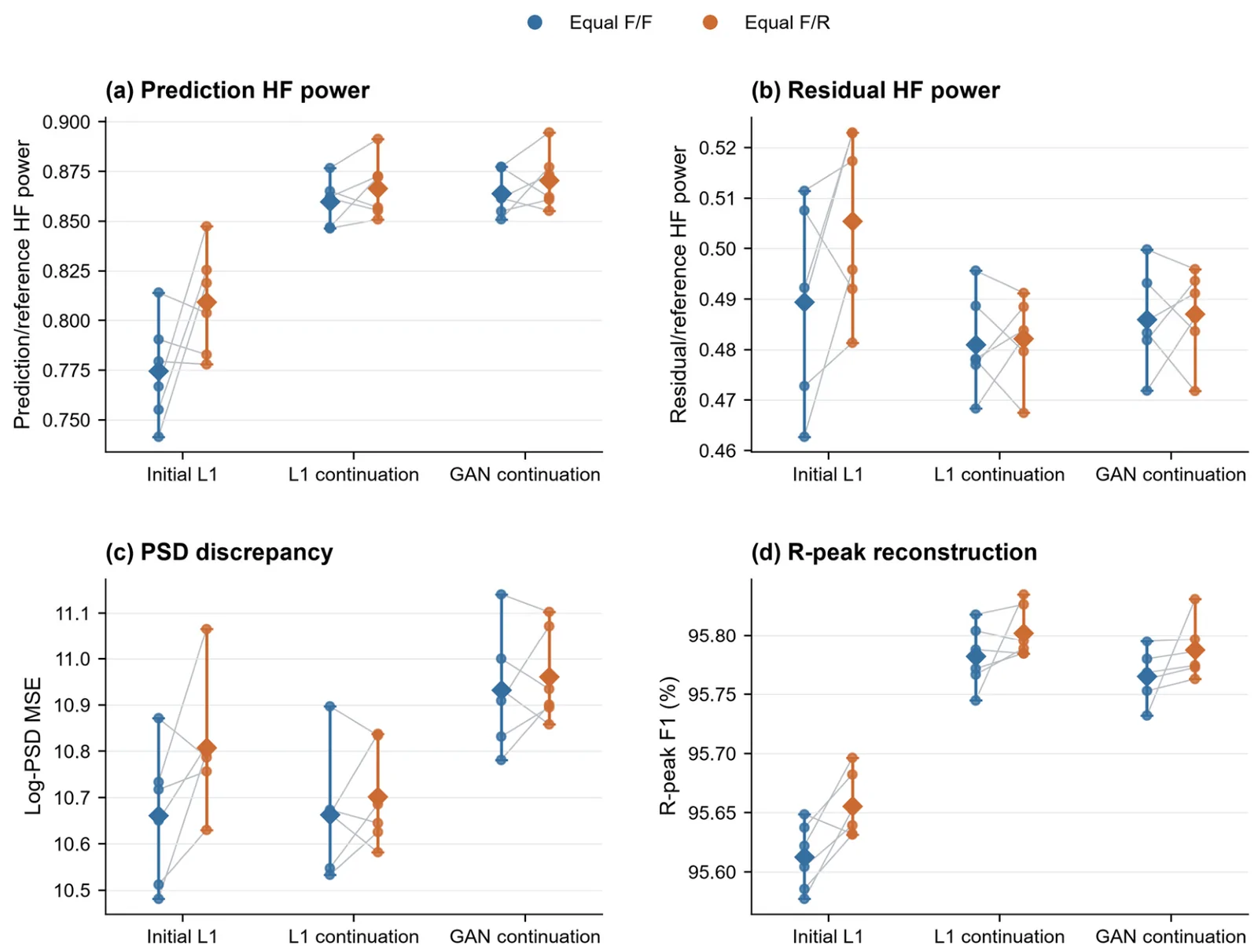
Spectral and peak reconstruction characteristics. Results for matched seed blocks 4–9 before and after continuation. Equal F/F and F/R are compared for (**a**) prediction/reference high-frequency power, (**b**) residual/reference high-frequency power, (**c**) log-PSD discrepancy and (**d**) R-peak *F*_1_.

Figure 5 compares the matched blocks 4–9 before and after continuation; Table 6 and Table 7 use all ten blocks for their initial-stage summaries. Small connected points show paired seeds, large markers show their means, and whiskers span minima and maxima. The HF ratios pool 40–100 Hz power within each seed’s cohort before summarizing across seeds. Log-PSD error covers the specified 0.5–100 Hz bins. R-peak *F*_1_ is displayed as a percentage and uses the 50 ms matching rule.

**Figure 6:**
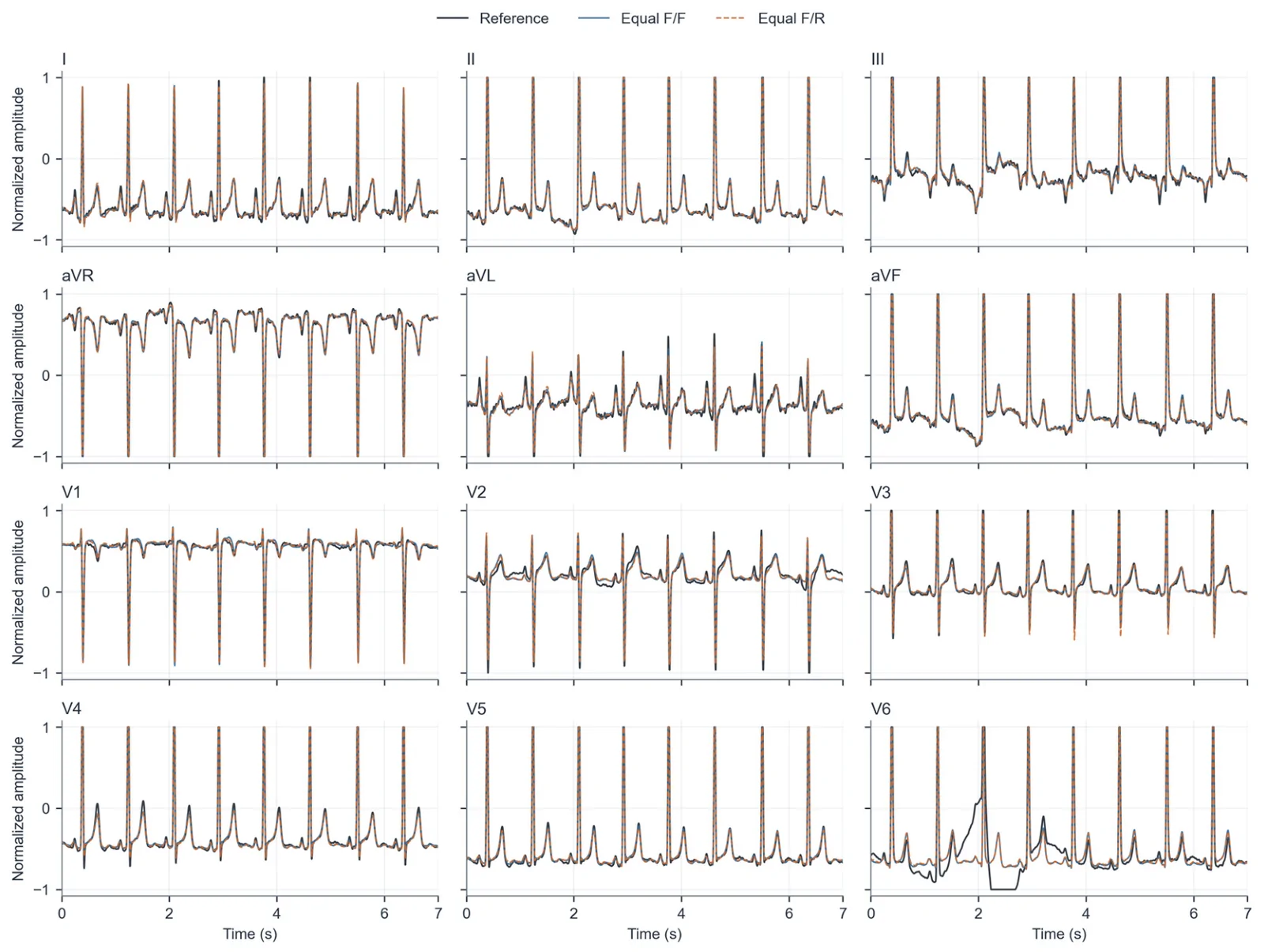
Twelve-lead waveform example after adversarial fine-tuning, showing the reference ECG and equal F/F and F/R reconstructions.

Figure 6 shows all twelve leads from the first saved test example for GAN seed block 4. Selection followed storage order and was independent of waveform quality or fusion improvement. Each panel includes all 2800 samples without display smoothing or downsampling. Reference, equal F/F and equal F/R waveforms appear in normalized units on the forward time axis, with the same vertical scale across leads. This record provides a waveform-level view of the reconstructions summarized by the aggregate measures.

### 4.6. Computational Cost

Each TCN contained 17,432,024 parameters. Equal F/F and F/R both used 34,864,048 parameters, while the local fusion head added 29,292 parameters. On an NVIDIA H100 PCIe, median inference latency for one seven-second, twelve-lead window was 1.244 ms for a single forward branch, 2.431 ms for equal F/F, 2.459 ms for equal F/R and 2.727 ms for local F/R. With a batch of 64 windows, the corresponding batch latencies were 8.670, 17.420, 17.753 and 18.836 ms. Equal F/F, equal F/R and local F/R achieved throughputs of 3674, 3605 and 3398 windows per second, respectively. Thus, equal directional fusion had inference costs closely matching those of ordinary forward fusion at the same capacity.

The 30 initial branch runs had a median recorded training-and-validation time of 133.7 min for 50 epochs, with a range of 133.0–136.2 min. The matched ten-epoch continuation runs had median times of 26.9 min for L1 and 29.0 min for GAN training. Each continuation branch received 16,940 generator updates. The GAN group additionally trained a 764,001-parameter discriminator with the same number of discriminator updates; this discriminator was removed at inference. The two continuation groups each comprised 18 branch runs, with summed recorded times of 8.06 h for L1 and 8.69 h for GAN training. The nine symmetric-padding branches had a median recorded time of 122.7 min per 50-epoch run and a summed recorded time of 18.41 h.

### 4.7. Padding and Boundary Controls

Boundary-excluded evaluation retained the mean directional benefit in the causal model. On test, equal F/R reduced mean MAE by 1.127% for the complete window and by 1.183% after excluding 400 samples from each end, with improvement in all three blocks. For symmetric padding, the corresponding mean reductions were 0.843% and 0.757%, with lower MAE in one of the three blocks (Table 8). Confirmation results showed the same mean direction. The positive mean reduction therefore extended into the interior of the window and was not solely a consequence of one-sided endpoint padding.

**Figure 7:**
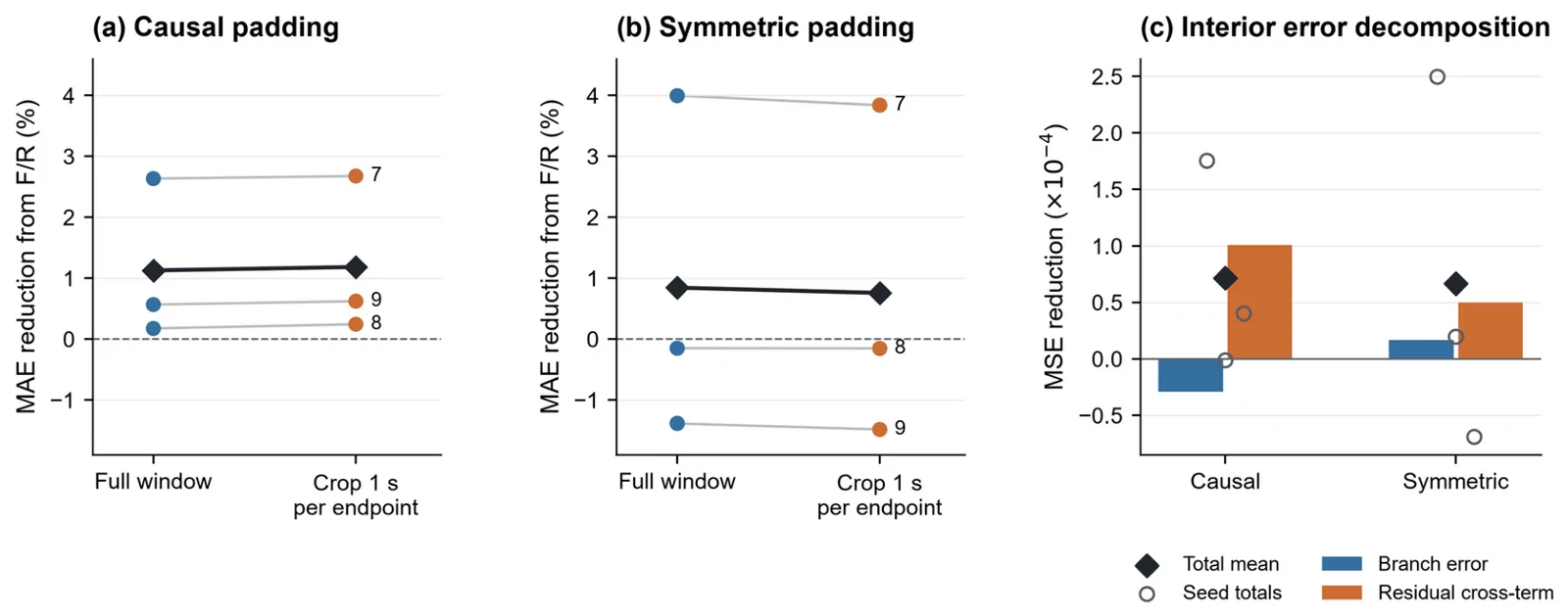
Directional fusion under (**a**) causal padding, comparing complete windows with excluding 400 samples at each end; (**b**) symmetric padding, comparing complete windows with excluding 400 samples at each end; (**c**) the interior MSE decomposition.

Figure 7 shows every seed-specific MAE reduction and the interior MSE decomposition. For the causal model after excluding 400 samples at each end, the mean MSE reduction was 0.000071375: a second-branch contribution of −0.000029369 was offset by a residual cross-term contribution of 0.000100744. The symmetric counterpart had a mean reduction of 0.000066705, comprising contributions of 0.000016706 and 0.000050000. These controls support computational directional complementarity beyond window endpoints, with greater seed variability under symmetric padding. Black diamonds in the MAE panels denote reductions calculated from the ratio of seed-averaged MAEs; the other markers show individual blocks. Crops and temporal bins provide paired evaluations within each trained-model replication.

## 5. Discussion

F/R fusion improved reconstruction beyond an ordinary two-forward ensemble. Equal weighting fixed the combination rule, and the identical branch architectures matched model capacity. Paired initialization further controlled the comparison between the second branches. Lower test MAE across ten initial seed blocks supports temporal direction as a useful source of reconstruction diversity. This benefit was more consistent in fusion than in the standalone reverse model.

The residual decomposition explains why branch-level accuracy alone does not determine fusion performance. Equal-fusion MSE depends on the cross-moment between errors as well as their individual magnitudes. Replacing *F*_B_ with *R*_B_ reduced this cross term and the fusion error, consistent with established ensemble analyses [18,42]. Although the branches were trained independently without a diversity penalty, changing temporal orientation produced errors that combined more favorably.

The results are consistent with complementary errors learned by a direction-sensitive architecture with a finite latent representation. The residual decomposition quantifies this complementarity without identifying a specific internal feature or optimization mechanism. Reversal is a controlled source of diversity because it preserves every input sample and lead while changing the temporal presentation learned by the model. The F/F baseline already includes diversity from independent initialization, and the paired forward and reverse second branches start from identical weights. This design separates direction from additional model capacity, training data or an augmentation that changes waveform values. The repeatable fusion difference and residual decomposition therefore describe computational complementarity, which is distinct from physiological time irreversibility [37,38].

The continuation experiment extends the finding to adversarial reconstruction. Generators received discriminator-guided updates, with reverse outputs aligned before real/fake evaluation. Equal F/R retained a lower MAE than equal F/F across all six evaluated GAN seed blocks, including all five nonpilot blocks, showing that the directional gain persisted after the change in training objective.

The matched *L*_1_ group controls for additional training. Both continuation groups reduced error relative to their starting models, with slightly lower MAE under the tested *L*_1_ configuration. Directional fusion improved reconstruction within each group. The comparison supports temporal diversity under both objectives and separates this benefit from the overall improvement during continuation.

Adaptive weighting further reduced MAE under the original backbone budget. Static and record-adaptive controls reduced MAE to a lesser extent than the local head, suggesting a benefit from allowing weights to vary during reconstruction. After both continuation protocols, development selection retained the equal-weight initialization for F/R. Equal directional fusion improved reconstruction within each continuation group, while the additional benefit of local weighting depended on the backbone training stage. The adaptive F/F control separates the effect of temporal direction from the general benefit of learning a combination rule [44].

The stress test assesses perturbations absent from backbone training and head fitting. Directional fusion improved seed-averaged GAN results across all prescribed conditions relative to an equally sized forward ensemble. The original stored recordings were the reference, and between-seed variability increased under the strongest corruption.

F/R reconstructions retained more HF power, with similar or higher HF residuals in the corresponding comparisons (Table 6). Mean R-peak agreement also increased (Table 7). The 0.717% mean MAE reduction after adversarial continuation quantifies a reconstruction gain relative to a capacity-matched ensemble. Clinical interpretation requires assessment of diagnostic features against expert annotations and clinical endpoints.

The findings concern the specified TCN and automatically normal-labeled CODE-15% cohort. Transfer to other model families, external clinical centers and ECGs with ST changes, conduction abnormalities or arrhythmias remains to be assessed. The adversarial extension contains six seed blocks and one development-selected fine-tuning configuration. Patient partitions were disjoint, but aggregate confirmation and test outcomes informed iterative method development. Repeated seed blocks assess training variability under the fixed protocol on these previously examined cohorts. The padding-control results indicate that repeatability also depends on architectural details and initialization.

It is worth noting that inputs were preprocessed as normalized windows, and the upstream amplitude transformation was not reconstructed from the archived arrays. The reported errors therefore cannot be converted directly to millivolts or compared with clinical amplitude tolerances. Fusion heads used in-sample backbone predictions, and their behavior may differ with a separate head-training cohort. Benefits varied across leads and became less uniform at the lowest SNR. Peak agreement used automatic detections; diagnostic preservation requires expert annotations or a separate clinical evaluation.

## 6. Conclusions

Time reversal fusion improved ECG reconstruction beyond a capacity-matched ensemble of two forward models. Equal F/R reduced mean test MAE by 1.437% under the initial training protocol, with lower MAE in all ten seed blocks. After adversarial fine-tuning, the mean reduction was 0.717%, with lower MAE in all six evaluated blocks. The residual decomposition linked the fusion gain to both the reverse branch’s own error and its lower error cross-moment with the shared forward branch. Temporal direction supplied complementary errors that improved fusion beyond the matched forward ensemble. The gain persisted in the interior of the reconstructed windows, and symmetric-padding controls retained a mean benefit.

Local adaptive F/R increased the initial reduction relative to equal F/F to 2.140%. Its added benefit depended on the training regime: after continuation, development selection retained equal F/R weights. Equal directional fusion also improved the seed-averaged adversarial results across all prescribed noise conditions, with greater variability under severe corruption. Spectral and peak analyses characterized retained HF power, residual error and timing agreement separately. Within the evaluated TCN and normal-labeled ECG cohort, these findings support time reversal as a practical source of ensemble diversity under both *L*_1_ and adversarial reconstruction objectives.

## Figures and Tables

**Table 1:** Reconstruction performance and fusion ablations under the initial *L*_1_ protocol.

Method	MAE	RMSE	Pearson *r*	R-Peak *F*_1_
Single *F*_A_	0.043876 ± 0.000335	0.071396 ± 0.000610	0.955941 ± 0.000730	0.954826 ± 0.000375
Single *F*_B_	0.043978 ± 0.000611	0.071415 ± 0.000998	0.955866 ± 0.001178	0.954876 ± 0.000525
Single *R*_B_	0.043780 ± 0.000592	0.071455 ± 0.000831	0.955891 ± 0.001029	0.955099 ± 0.000467
Equal F/F	0.041493 ± 0.000298	0.067536 ± 0.000575	0.960503 ± 0.000660	0.956151 ± 0.000333
Equal F/R	0.040897 ± 0.000305	0.066772 ± 0.000410	0.961300 ± 0.000467	0.956620 ± 0.000282
Static F/F	0.041456 ± 0.000282	0.067497 ± 0.000565	0.960530 ± 0.000646	0.956134 ± 0.000350
Static F/R	0.040869 ± 0.000313	0.066743 ± 0.000416	0.961314 ± 0.000478	0.956617 ± 0.000272
Record-adaptive F/F	0.041431 ± 0.000281	0.067477 ± 0.000556	0.960515 ± 0.000626	0.956163 ± 0.000350
Record-adaptive F/R	0.040847 ± 0.000306	0.066723 ± 0.000413	0.961311 ± 0.000480	0.956621 ± 0.000263
Local-adaptive F/F	0.041153 ± 0.000297	0.067174 ± 0.000577	0.960609 ± 0.000642	0.955862 ± 0.000473
Local-adaptive F/R	0.040605 ± 0.000262	0.066461 ± 0.000370	0.961422 ± 0.000450	0.956510 ± 0.000295

**Table 2:** Paired primary MAE comparisons under the initial protocol.

Seed Blocks	Comparison	Δ MAE × 10^−4^	95% CI × 10^−4^	Relative Change	Lower MAE	Holm *p*
1–10	Local F/R − Equal F/F	−8.878	[−11.801, −5.954]	−2.140%	10/10	0.005859
1–10	Local F/R − Local F/F	−5.475	[−8.282, −2.669]	−1.331%	10/10	0.005859
1–10	Local F/R − Equal F/R	−2.915	[−3.481, −2.349]	−0.713%	10/10	0.005859
4–10	Local F/R − Equal F/F	−9.095	[−13.147, −5.043]	−2.187%	7/7	0.046875
4–10	Local F/R − Local F/F	−5.399	[−9.242, −1.556]	−1.310%	7/7	0.046875
4–10	Local F/R − Equal F/R	−3.083	[−3.733, −2.432]	−0.752%	7/7	0.046875

**Table 3:** Reconstruction performance after matched continuation.

Training	Method	MAE	RMSE	Pearson *r*	R-Peak *F*_1_
*L*_1_	Single *F*_A_	0.041344 ± 0.000396	0.067566 ± 0.000516	0.959777 ± 0.000643	0.956445 ± 0.000263
*L*_1_	Single *F*_B_	0.041486 ± 0.000409	0.067634 ± 0.000551	0.959698 ± 0.000733	0.956659 ± 0.000276
*L*_1_	Single *R*_B_	0.041328 ± 0.000380	0.067621 ± 0.000730	0.959747 ± 0.000803	0.956825 ± 0.000236
*L*_1_	Equal F/F	0.039418 ± 0.000226	0.064519 ± 0.000379	0.963417 ± 0.000365	0.957821 ± 0.000267
*L*_1_	Equal F/R	0.039148 ± 0.000277	0.064212 ± 0.000465	0.963786 ± 0.000493	0.958024 ± 0.000225
*L*_1_	Local-adaptive F/F	0.039418 ± 0.000226	0.064519 ± 0.000379	0.963416 ± 0.000365	0.957810 ± 0.000261
*L*_1_	Local-adaptive F/R	0.039148 ± 0.000277	0.064212 ± 0.000465	0.963786 ± 0.000493	0.958024 ± 0.000225
GAN	Single *F*_A_	0.041562 ± 0.000393	0.067774 ± 0.000543	0.959493 ± 0.000674	0.956244 ± 0.000196
GAN	Single *F*_B_	0.041698 ± 0.000414	0.067824 ± 0.000546	0.959442 ± 0.000723	0.956474 ± 0.000278
GAN	Single *R*_B_	0.041537 ± 0.000356	0.067805 ± 0.000716	0.959504 ± 0.000791	0.956760 ± 0.000195
GAN	Equal F/F	0.039523 ± 0.000234	0.064590 ± 0.000399	0.963307 ± 0.000386	0.957651 ± 0.000218
GAN	Equal F/R	0.039239 ± 0.000267	0.064265 ± 0.000468	0.963698 ± 0.000501	0.957873 ± 0.000244
GAN	Local-adaptive F/F	0.039519 ± 0.000241	0.064595 ± 0.000388	0.963296 ± 0.000363	0.957643 ± 0.000206
GAN	Local-adaptive F/R	0.039239 ± 0.000267	0.064265 ± 0.000468	0.963698 ± 0.000501	0.957873 ± 0.000244

**Table 4:** Descriptive MAE effects after continuation.

Treatment	Comparison	Δ MAE ± SD × 10^−4^	Relative Change	Lower MAE	Equal Seeds
GAN	Equal F/R − Equal F/F	−2.835 ± 1.088	−0.717%	6/6	0
GAN	Local F/R − Local F/F	−2.794 ± 1.063	−0.707%	6/6	0
GAN	Local F/R − Equal F/R	0.000 ± 0.000	0.000%	0/6	6
*L*_1_	Equal F/R − Equal F/F	−2.703 ± 1.049	−0.686%	6/6	0
*L*_1_	Local F/R − Local F/F	−2.702 ± 1.049	−0.686%	6/6	0
*L*_1_	Local F/R − Equal F/R	0.000 ± 0.000	0.000%	0/6	6
GAN − *L*_1_	Equal F/F	1.047 ± 0.155	+0.266%	0/6	0
GAN − *L*_1_	Equal F/R	0.915 ± 0.107	+0.234%	0/6	0

**Table 5:** Direction, adaptation and adversarial-continuation effects under controlled noise.

Noise	SNR (dB)	Initial F/R − F/F	Initial Local − Equal F/R	GAN F/R − F/F	GAN − *L*_1_ Equal F/R
Original input	N/A	−1.363% (9/10)	−0.740% (10/10)	−0.659% (6/6)	+0.244% (0/6)
Baseline wander	24	−1.390% (10/10)	−0.711% (10/10)	−0.715% (6/6)	+0.241% (0/6)
Baseline wander	18	−1.457% (10/10)	−0.626% (10/10)	−0.862% (6/6)	+0.236% (0/6)
Baseline wander	12	−1.629% (10/10)	−0.330% (10/10)	−1.234% (5/6)	+0.223% (0/6)
Baseline wander	6	−1.783% (9/10)	+0.585% (0/10)	−1.870% (5/6)	+0.213% (0/6)
Baseline wander	0	−2.259% (10/10)	+3.337% (0/10)	−2.912% (5/6)	+0.216% (0/6)
Baseline wander	−6	−3.499% (7/10)	+11.391% (0/10)	−4.228% (4/6)	+0.106% (4/6)
Muscle artifact	24	−1.382% (9/10)	−0.716% (10/10)	−0.692% (6/6)	+0.242% (0/6)
Muscle artifact	18	−1.437% (10/10)	−0.649% (10/10)	−0.804% (6/6)	+0.237% (0/6)
Muscle artifact	12	−1.586% (10/10)	−0.405% (10/10)	−1.143% (5/6)	+0.225% (0/6)
Muscle artifact	6	−1.681% (10/10)	+0.442% (0/10)	−1.775% (5/6)	+0.216% (0/6)
Muscle artifact	0	−1.663% (6/10)	+3.324% (0/10)	−2.205% (4/6)	+0.242% (0/6)
Muscle artifact	−6	−2.496% (5/10)	+12.161% (0/10)	−2.580% (3/6)	+0.132% (2/6)
Electrode motion	24	−1.389% (9/10)	−0.700% (10/10)	−0.706% (6/6)	+0.241% (0/6)
Electrode motion	18	−1.457% (10/10)	−0.587% (10/10)	−0.828% (6/6)	+0.234% (0/6)
Electrode motion	12	−1.660% (10/10)	−0.198% (10/10)	−1.159% (6/6)	+0.218% (0/6)
Electrode motion	6	−1.903% (10/10)	+1.022% (0/10)	−1.802% (5/6)	+0.206% (0/6)
Electrode motion	0	−1.971% (7/10)	+5.026% (0/10)	−2.112% (5/6)	+0.171% (0/6)
Electrode motion	−6	−1.511% (6/10)	+14.013% (0/10)	−1.438% (4/6)	−0.041% (5/6)

**Table 6:** Spectral fidelity across training protocols.

Protocol	Method	HF Power Ratio	HF Error Ratio	PSD Log-MSE	HFRR
Initial *L*_1_ (*n* = 10)	Equal F/F	0.793657 ± 0.039073	0.507385 ± 0.033895	10.763210 ± 0.249123	0.057807 ± 0.001263
Initial *L*_1_ (*n* = 10)	Equal F/R	0.814751 ± 0.024715	0.510848 ± 0.017915	10.827846 ± 0.153609	0.057293 ± 0.000686
Initial *L*_1_ (*n* = 10)	Local-adaptive F/F	0.846841 ± 0.019549	0.530397 ± 0.023601	11.082327 ± 0.234841	0.057413 ± 0.001076
Initial *L*_1_ (*n* = 10)	Local-adaptive F/R	0.869847 ± 0.017371	0.531382 ± 0.012945	11.101192 ± 0.095404	0.056787 ± 0.000532
*L*_1_ (*n* = 6)	Equal F/F	0.859781 ± 0.011557	0.480938 ± 0.009658	10.663110 ± 0.130470	0.052805 ± 0.000404
*L*_1_ (*n* = 6)	Equal F/R	0.866559 ± 0.015153	0.482172 ± 0.008332	10.701651 ± 0.109237	0.052631 ± 0.000452
GAN (*n* = 6)	Equal F/F	0.863914 ± 0.011076	0.485955 ± 0.009657	10.932674 ± 0.127644	0.052967 ± 0.000387
GAN (*n* = 6)	Equal F/R	0.870552 ± 0.014371	0.487049 ± 0.008758	10.960397 ± 0.101677	0.052794 ± 0.000438

**Table 7:** R-peak reconstruction across training protocols.

Protocol	Method	Precision	Recall	*F*_1_	Matched Peaks
Initial *L*_1_ (*n* = 10)	Equal F/F	0.980523 ± 0.001955	0.940903 ± 0.001750	0.956151 ± 0.000333	9.177581 ± 0.021782
Initial *L*_1_ (*n* = 10)	Equal F/R	0.980433 ± 0.001001	0.941702 ± 0.000988	0.956620 ± 0.000282	9.189453 ± 0.012795
Initial *L*_1_ (*n* = 10)	Local-adaptive F/F	0.979003 ± 0.001800	0.941588 ± 0.001477	0.955862 ± 0.000473	9.185923 ± 0.018277
Initial *L*_1_ (*n* = 10)	Local-adaptive F/R	0.979391 ± 0.000776	0.942322 ± 0.000756	0.956510 ± 0.000295	9.196112 ± 0.009745
*L*_1_ (*n* = 6)	Equal F/F	0.980640 ± 0.000509	0.943636 ± 0.000464	0.957821 ± 0.000267	9.213380 ± 0.005514
*L*_1_ (*n* = 6)	Equal F/R	0.980820 ± 0.000238	0.943810 ± 0.000419	0.958024 ± 0.000225	9.216346 ± 0.005536
GAN (*n* = 6)	Equal F/F	0.980549 ± 0.000444	0.943411 ± 0.000548	0.957651 ± 0.000218	9.210003 ± 0.006687
GAN (*n* = 6)	Equal F/R	0.980636 ± 0.000243	0.943687 ± 0.000408	0.957873 ± 0.000244	9.214435 ± 0.005621

**Table 8:** Test MAE under matched causal and symmetric padding, with evaluation-only exclusion of boundary samples.

Padding	Crop per End	F/F MAE	F/R MAE	Reduction	Improved
Causal	0	0.041424	0.040957	1.127%	3/3
Causal	100	0.041048	0.040582	1.135%	3/3
Causal	200	0.040860	0.040388	1.156%	3/3
Causal	400	0.040520	0.040041	1.183%	3/3
Symmetric	0	0.041606	0.041255	0.843%	1/3
Symmetric	100	0.041261	0.040914	0.840%	1/3
Symmetric	200	0.041050	0.040717	0.811%	1/3
Symmetric	400	0.040686	0.040378	0.757%	1/3

## Data Availability

The CODE-15% dataset used in this study is publicly available and can be accessed through the Zenodo repository (https://doi.org/10.5281/zenodo.4916206).

## Abbreviations

The following abbreviations are used in this manuscript:

BW	Baseline wander
CI	Confidence interval
ECG	Electrocardiogram
EM	Electrode motion
F/F	Forward/forward fusion
F/R	Forward/reverse fusion
GAN	Generative adversarial network
GELU	Gaussian error linear unit
HF	High frequency
HFRR	High-frequency residual ratio
LeakyReLU	Leaky rectified linear unit
LSGAN	Least-squares generative adversarial network
MA	Muscle artifact
MAE	Mean absolute error
MSE	Mean squared error
NSTDB	MIT-BIH Noise Stress Test Database
PSD	Power spectral density
RMS	Root mean square
RMSE	Root mean squared error
rPPG	Remote photoplethysmography
SD	Standard deviation
SNR	Signal-to-noise ratio
TCN	Temporal convolutional network
